# Tremendous Acceleration of Plant Growth by Applying a New Sunlight Converter Sr_4_Al_14−_ *_x_* Ga*_x_* O_25_:Mn^4+^ Breaking Parity Forbidden Transition

**DOI:** 10.1002/advs.202204418

**Published:** 2022-11-24

**Authors:** Shichuan Wang, Takatoshi Seto, Bin Liu, Yuhua Wang, Cancan Li, Zhengqiang Liu, Haowen Dong

**Affiliations:** ^1^ National and Local Joint Engineering Laboratory for Optical Conversion Materials and Technology of National Development and Reform Commission School of Materials and Energy Lanzhou University Lanzhou 730000 China

**Keywords:** breaking parity forbidden, oxide phosphor, plant cultivation, red emission phosphor, sunlight converter

## Abstract

Majority of Mn^4+^ activated oxide phosphors have the wavelength of excitation and emission suitable for acceleration of plant growth as light converter from sunlight to deep red. Here, it is observed that 60% increase of red emission of Sr_4_Al_14_O_25_:0.01Mn^4+^ is found by substituting 0.1Ga^3+^. It is clarified that the increase is originated from a unique mechanism of breaking parity forbidden transition under the substitution of cation in d–d transition by using the tool of special aberration corrected transmission electron microscope(AC‐STEM), pre‐edge peak (1s→3d) Mn K‐edge X‐ray absorption near edge structure (XANES), extended X‐ray absorption fine structure (EXAFS), Rietveld analysis of X‐ray diffraction (XRD) patterns, and reflection spectra. Further, a combination of substituted Ga, Mg, and special double flux H_3_BO_3_/AlF_3_ is found to tremendously increase the emission intensity (355% up). Actual growth of chlorella and rose is examined by a combination of the cheap Sr_4_Al_14_O_25_:0.01Mn^4+^,0.007Mg^2+^,0.1Ga^3+^ and a unique reflection typed phosphor‐film system as sunlight converting system. Optical density of chlorella and height of rose grass is increased by 36±14% and 174±80% compared with nonphosphor‐film, respectively.

## Graphical abstract

An oxide phosphor for plant growth with excellent luminescence is prepared by changing the local structure of the luminescence center by Ga ion substitution method and breaking the mechanism of parity prohibition of conversion. And a new type of highly efficient reflective light conversion film is prepared by mixing it with PDMS to promote plant growth.

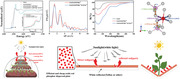

## 1 Introduction

Nowadays, with the improvement of people's living standard, people's demand for healthy living and green environment is increasing day by day, while traditional methods of increasing yield of crops through chemical fertilizers and pesticides cannot meet these demands because they produce a lot of pollution. So, the way of enhancing plant growth efficiency through fluorescent conversion materials has been proposed and widely studied and discussed.

In general, sunlight plays a vital role in plant growth, and three specific wavelengths of light are particularly important for plant growth: blue light (400–500 nm), red light (620–690 nm), and near‐infrared color (730–760 nm), which are responsible for phototropism,^[^ ^1^ ^]^ photosynthesis,^[^ ^2^ ^]^ and photomorphogenesis,^[^ ^3^ ^]^ respectively. Among them, photosynthesis is a necessary condition for plant growth, so enhancing the light intensity in the red band can effectively enhance the photosynthesis of plants. However, the ratio of the red part in total sunlight is very small, resulting in the low absorption efficiency of plants in this band, therefore, red fluorescent conversion materials with excellent performance are needed to compensate for it. LED‐induced plant growth has been proposed^[^ ^4^ ^]^ and successfully contributed to the scientific clarification of the relation between the actual growth of each specific crop and each specific wavelength of light for more than two decades, while its business and industry is not thriving due to its highly costing facilities, LED devices, and electricity, etc.

**Figure 1:**
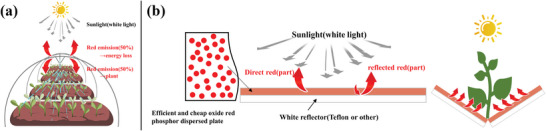
Plant growth method of a) emitting materials dispersed greenhouse, and b) our new method “reflection‐type red emitting phosphor‐plate under sunlight”.

Compared with LED plant growth factory, a relatively low costing method is a method that the red or far red light yielded from sunlight through emitting film is exposure to the plants.^[^ ^5^ ^]^ Because the latter does not need highly costing facilities, LED devices, and electricity, etc. Majority of the latter method has been greenhouse type as shown in **Figure** **1**a, where emitting powders dispersed resin film surrounds the plants or crops, which can receive the red or far red light emitted from the film with low cost. The fundamental problem in this greenhouse type is that red light spread towards not only inside the greenhouse but also outside the greenhouse in principle, which causes just 50% loss of the red light for plant growth. Technologies of modifying distribution or size of emitting powders in the film improves it to only (50 – several) %.^[^ ^6^ ^]^

We construct a method of reflection typed phosphor‐plate nearby plant where sunlight is coming to the phosphor‐plate having thin white reflector on back‐side, yielding red light whose majority can be directed to plant as shown in Figure 1b. At first 50% of red light from phosphor‐powders is directed towards the side opposite plants, then being returned to the side of plants by the existence of white reflector. Theoretical flux of red emission which plants can receive in the reflection type is 1.4–2 times of greenhouse type. The new system has much higher ability of giving the sunlight‐converted red light to the plant. Typical phosphor converting sunlight to red light could be CaAlSiN_3_:Eu, which is the best NUV and blue excited, red emission phosphor for LED lighting. However, CaAlSiN_3_:Eu is highly costing (cost of CaAlSiN_3_:Eu: oxide phosphors = 5–8: 1), compared with cheap crops or plants due to its severe batch styled synthesis at about 1800 °C under high pressure and highly costing nitride starting materials such as Ca_3_N_2_.

For the subject of creating low costing phosphors, we have considered not nitride phosphor but oxide phosphor in recent years. In oxide phosphors activated by Eu^2+^ or Ce^3+^, relatively strong excitation and emission are often obtained due to their parity and spin allowed 4f→5d transition, while these are likely to provide limited wavelength of emission, not reaching red emission of 650–685 nm important for plant growth due to relatively low crystal field splitting and nephelauxetic effect in [EuO*_x_*] and [CeO*_x_*] polyhedron, compared with [EuN*_x_*] in CaAlSiN_3_:Eu. In oxide phosphors activated by Mn^4+^ are, in many cases, stable, nontoxic, and provides fine red emission of 650–685 nm due to the relatively small energy difference between excited ^2^E state and ground ^4^A_2_ state caused by relatively large Racar B value and small nephelauxetic effect, compared with toxic, not much stable fluoride phosphors activated by Mn^4+^ such as K_2_SiF_4_:Mn^4+^ (KSF), famous Mn^4+^‐phosphor having 630 nm emission for LED display, in Tanabe–Sugano d^3^ energy diagram. However, in oxide phosphors activated by Mn^4+^, the excitation and emission are significantly small in most cases due to parity forbidden 3d→3d transition of ^4^A_2_→4T_1_, ^4^A_2_→^4^T_2_ (Ex.), and ^2^E→^4^A_2_ (Em.). For solving this problem, we have struggled with local structure having this parity forbidden property. Here, we would like to present a successful break of parity forbidden transition in stable phosphor Sr_4_Al_14_O_25_:Mn^4+^ having good NUV and blue excitation, and good emission, 653 nm (red) for plant growth. We exhibit the surprising result of actual chlorella and rose growth too by using the above reflection typed plate having the Mn^4+^ activated oxide phosphor where parity forbidden is broken.

## 2 Experimental Section

### 2.1 Synthesis of Materials

#### 2.1.1 Preparation of Phosphor

Mn^4+^,Ga^3+^‐substituted Sr_4_Al_14_O_25_ (SAO) phosphor was prepared by high‐temperature solid‐state method. The raw materials were SrCO_3_ [analytical reagent (A.R.)], Al_2_O_3_ (99.99%), Ga_2_O_3_ (99.99%), and MnO_2_ (99.99%); the raw materials were weighed according to certain stoichiometric ratios, then uniformly mixed and finely ground in an agate mortar. The mixture was then packed in an alumina crucible and sintered in a high temperature tube furnace under air atmosphere at 1260 °C for 6 h. After cooling to room temperature, the samples were ground again for further measurements. SAO: Mn^4+^, Mg^2+^, Z^3+^ (Z^3+^: Ga^3+^, Sc^3+^, or Lu^3+^) was also prepared by using the above raw materials, MgCO_3_ (99.99%), Sc_2_O_3_ (99.99%), or Lu_2_O_3_ (99.99%) in the above condition. H_3_BO_3_ (99.99%) and AlF_3_ (99.99%) were also used as flux.

#### 2.1.2 Preparation of Phototransfer Film

The red phosphor SAO: Mn^4+^, Mg^2+^, Ga^3+^, and adhesive of PDMS were mixed in proportion, stirred in a vacuum defoamer for 3 min, and then evenly coated in the grooves of a 30 cm × 30 cm × 2 mm glass plate. After that it was placed in an oven at 60 °C for 6 h, dried and removed to obtain the light conversion film. Teflon thin film was set on back side of SAO: Mn^4+^, Mg^2+^, Ga^3+^ film as a white reflector of light.

## 3 Characterization

The phase purity of the prepared aluminates was analyzed by powder X‐ray diffraction (XRD) on a RigakuD/max‐2400 X‐ray diffractometer with K*α* radiation. XRD patterns were acquired in the range of 10°–80° with a counting time of 0.1 s per step and a step size of 0.03°. Rietveld was modified using the General Structural Analysis System (GSAS)). The morphology was obtained by scanning electron microscopy (SEM, S‐3400, Hitachi, Japan) and transmission electron microscopy (TEM)). An EDX detector attached to the same SEM was used to explore the elemental composition. High resolution transmission electron microscopy (HRTEM) was performed with a FEI TecnaiF30TEM at 300KV. Photoluminescence excitation (PLE) and photoluminescence (PL) spectra were collected using an Edinburgh Instruments FLS920. Quantum efficiency was measured with the quantum yield attachment attached to the FLS‐920T fluorescence spectrophotometer. PL decay curves were collected with the FLS‐920T fluorescence spectrophotometer. Thermal burst performance was measured with an aluminum patch with a cartridge heater; temperature was measured with a thermocouple inside the patch and controlled by a standard TAP‐02 high‐temperature fluorescence controller (Oriental KOJI Co., Ltd., Tianjin, China). Spherical aberration‐corrected transmission electron microscope (AC‐STEM), JEM‐ARM200F, was used for confirming the structure and detecting the position of impurities such as Ga. Mn K edge XAFS was measured at the beam‐line of Shanghai XAFS Institute.

## 4 Results and Discussion

### 4.1 Fundamental Analysis of Structure and Optics

**Figure 2:**
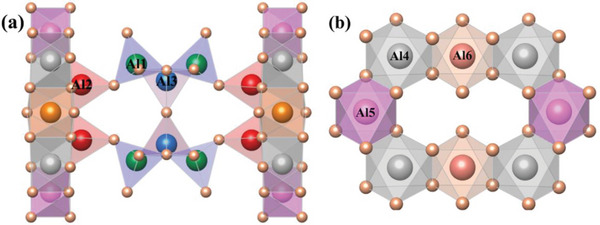
a) SAO crystal structure diagram and b) [AlO] polyhedral ring structure diagram.

**Figure** **2**a shows the crystal structure of SAO. The SAO sample space group belongs to the centrosymmetric space group Pmma (51), and the crystal system is orthogonal. The lattice consists of three [AlO_4_] tetrahedra, three [AlO_6_] octahedra, and [SrO_7_] and [SrO_10_] polyhedra, in which [AlO_4_], [Al_2_O_4_], and [Al_3_O_4_] are codotted and arranged alternately in the *a*‐direction; while [Al_4_O_4_], [Al_5_O_4_], and [Al_6_O_4_] are connected to each other by coprismatic connection to form the ring structure, as shown in Figure 1b; the [AlO_4_] tetrahedra and [AlO_6_] octahedra form an ordered lamellar structure by codot connections between them.

Based on the previous analysis, we prepared SAO:*x*Mn^4+^ and tested its emission spectrum (Figure S1, Supporting Information), yielding optimal emission intensity at 0.01Mn^4+^ and characterized the luminescence properties of a series of samples codoped with Mn^4+^ and Ga^3+^ and characterized them, and measured its series of XRD patterns and decay, as shown in Figures S2 and S9 in the Supporting Information. Sr_4_Al_14−_ *_x_* Ga*_x_* O_25_ Rietveld diagram is shown in Figure S3 in the Supporting Information.

**Figure 3:**
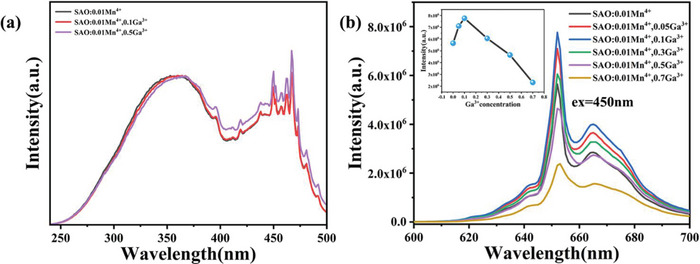
a) Normalized excitation and b) emission spectra of SAO:0.01Mn^4+^/*x*Ga^3+^ (0 ≤ *x* ≤ 0.7).

**Figure** **3** shows normalized excitation and emission spectra of SAO:0.01Mn^4+^/*x*Ga^3+^ (0 ≤ *x* ≤ 0.7) samples. The excitation spectra mainly consist of two peaks located at NUV region and blue region, which are attributed to spin allowed ^4^A_2_→^4^T_1_ and ^4^A_2_→^4^T_2_ transitions, respectively.^[^ ^7^ ^]^ Trivial contribution to excitation is O^2−^→Mn^4+^ charge transfer (minimum wavelength) and spin forbidden ^4^A_2_→^2^T_2_ (a little longer wavelength than that of ^4^A_2_→4T_1_).^[^ ^8^ ^]^ The emission spectra is assigned to a sharp peak centered at 653 nm and a broad band located at 665 nm, which correspond to the ^2^E→^4^A_2_ transition of Mn^4+^ and a phonon assisted sideband, respectively.^[^ ^9^ ^]^

It was observed that the emission intensity significantly increases with increasing Ga^3+^ up to *x* = 0.1, then it decreases due to concentration quenching. We found the 60% increase of the emission intensity by substituting 0.1Ga(EQE = 31.61%,as shown in Table S3 in the Supporting Information). Transmission electron microscopy tests and EDS tests were performed on this sample, as shown in Figure S6 in the Supporting Information.

**Figure 4:**
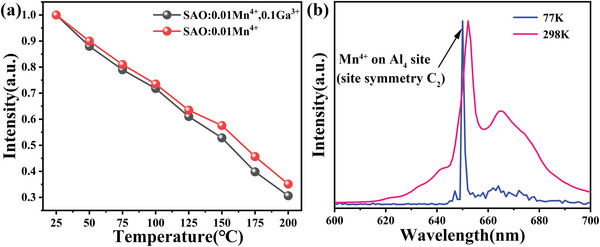
a) Temperature dependency of emission of SAO:0.01Mn^4+^ and SAO:0.01Mn^4+^,0.1Ga^3+^, b) emission spectra of SAO:Mn^4+^ at room temperature and 77 K.

Substitution of new cation often improves thermal stability, namely, temperature dependency of emission, simultaneously improving the emission intensity. Bad temperature dependency of emission relates to the next two mechanism.^[^ ^10^ ^]^ No.1. Auto‐ionization, where photoexcited electron at 5d band jumps to conduction band with help of heat of room temperature,^[^ ^11^ ^]^ and No.2. nonemissive process in configuration coordinate, where photoexcited electron jumps to the intersection between the ground‐state curve and the excited‐state curve.^[^ ^12^ ^]^ Bad emission induced by No.1 mechanism has nothing to do with defects. Bad emission induced by No.2 mechanism is considered to be only partly influenced by defects. So, the substitution of new cation often depresses No.1 or No.2 phenomenon, improving emission intensity even without depressing defects.^[^ ^13^ ^]^ We investigated the temperature dependency of emission of SAO:Mn^4+^,Ga^3+^, substitution of Ga^3+^ does not improve the temperature dependency of emission, which is shown in **Figure** **4**a (Figure S5 in the Supporting Information for details). Therefore, the reason for much increase of emission intensity by the substitution of Ga^3+^ is not the depression of auto‐ionization or nonemissive process in configurational coordinate.^[^ ^14^ ^]^

Figure 4b shows the emission spectra of Sr_4_Al_14_O_25_:Mn^4+^ at 77 K. A strong sharp emission at 652 nm is assigned to zero phonon line ^2^E→^4^A_2_ transition of Mn^4+^ on Al_4_ site (site symmetry in O_h_: C_2_) havig no inversion symmetry, while very small sharp emission at 647 nm is considered zero phonon line ^2^E→^4^A_2_ transition of Mn^4+^ on Al_5_ or Al_6_ site (site symmetry in O_h_: C_2h_) having inversion symmetry.^[^ ^15^ ^]^ Inversion symmetry in octahedron causes parity forbidden property at 3d→3d transition, exhibiting much small emission, while noninversion symmetry in octahedron more or less mitigates parity forbidden property at 3d→3d transition, exhibiting relatively stronger emission. Peng et al. calculated the average covalency of Al–O bonds on Al_4_, Al_5_, and Al_6_ sites by using the dielectric chemical bond theory of complex ionic crystals. The result was the covalency: Al_4_ > Al_5_, Al_6_, which indicates that Mn^4+^ on Al_4_ site has larger covalency and smaller Racah B value (B_R_) than Mn^4+^ on A_l5_ or Al_6_ site. As the energy difference of ^2^E and ^4^A_2_ is nearly proportional to B_R_ or √(B_R_/B_R0_)^2^ + (C_R_/C_R0_)^2^ (C_R_: Racah C value)^[^ ^16^ ^]^ in 3d^3^ configurational Tanabe–Sugano energy diagram, the energy difference is slightly smaller in Mn^4+^/Al_4_ site than in Mn^4+^/A_5_ or Mn^4+^/Al_6_ site, which coincides with the wavelength of the assigned zero phonon line: Mn^4+^/Al_4_ site > Mn^4+^/Al_5_ or Al_6_ site in Figure 4b. From the above information and consideration, it is reasonable that the important two emission of 652 nm (zero phonon line) and 665 nm (phonon assisted side band much enhanced at room temperature) are originated from Mn^4+^ on Al_4_ site. In Figure 3a, there is no significant change in peak‐wavelength in the main excitation of NUV (^4^A_2_→^4^T_1_) and blue (^4^A_2_→^4^T_2_) in case of substitution of Ga^3+^, which indicates that crystal field splitting (Cfs) of Mn^4+^ emitting red is not significantly changed by substituting Ga^3+^. ^4^T_2_ and ^4^T_1_ are in e_g_ level and ^4^A_2_ is in t_2g_ level in 3d^3^ configurational Tanabe–Sugano energy diagram. If Cfs of Mn^4+^ (O_h_) were changed, energy differences between ^4^A_2_ and ^4^T_2_ and between ^4^A_2_ and ^4^T_1_ should be changed. No significant change of Cfs is supported by the result of our Rietveld analysis that average bond length *R* of 6(Mn/Al)‐O at Al_4_ site relating to Cfs of Mn^4+^ (O_h_) is not much different, 1.911 Å (Ga molar ratio: 0) and 1.904 Å (Ga molar ratio: 0.1) because Cfs is proportional to *R* ^−5^ (from simple point charge approximation)^[^ ^16^ ^]^ or *R* ^−^ *^n^* (*n*: 3.5–6).^[^ ^17^ ^]^

### 4.2 AC‐STEM

**Figure 5:**
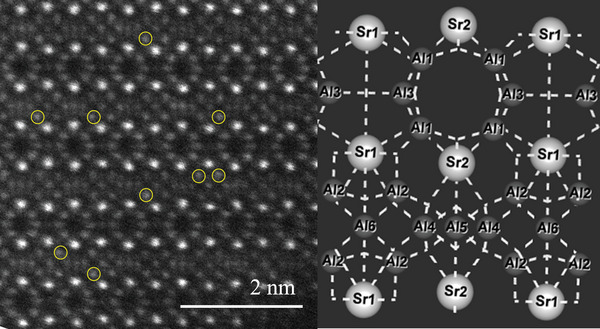
AC‐STEM image of SAO:0.01Mn^4+^,0.7Ga^3+^.

For the purpose of searching out the reason for emission increase, a spherical aberration‐corrected transmission electron microscope (AC‐STEM) of SAO:0.01Mn^4+^/0.7Ga^3+^ was Measured, and the results are shown in **Figure** **5**.

The STEM photograph shown in the figure is the atomic arrangement of the *a–b* plain, where it can be clearly seen that the larger bright white dots are Sr atoms, and the smaller dark gray dots are Al atoms in six different sites. Some of the dots corresponding to the Al_2_ site are brighter than the other Al sites (marked by the yellow circles), while this phenomenon is not found at Al sites other than Al_2_ site. Since atoms with larger atomic number show higher brightness in AC‐TEM images,^[^ ^18^ ^]^ it is strongly considered that the brighter atoms in the Al_2_ site mentioned above are Ga. This is supported by the results of Rietveld analysis of Sr_4_Al_13.9_Ga_0.3_O_25_ that *χ* ^2^ in case of Ga at Al_1_, Al_2_, Al_3_, Al_4_, Al_5_, and Al_6_ sites is 2.418, 2.223 (minimum), 2.449, 2.386, 2.461, and 2.257, respectively, and *R*_wp_ and *R*_b_ values in case of Ga at Al_2_ site are also in minimum level in 6 values.

### 4.3 XAFS

**Figure 6:**
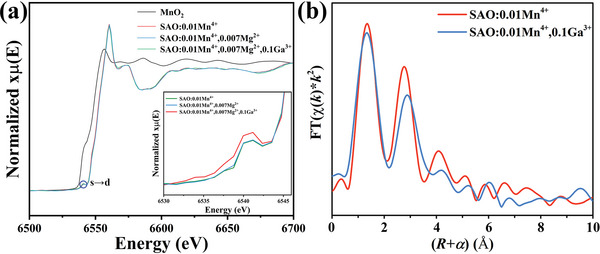
a) Mn K edge XAFS curves of SAO:Mn^4+^ and SAO:Mn^4+^,Ga^3+^. XANES (left side) and EXAFS (right side), and b) Mn K‐edge EXAFS‐Fourier transform showing *R*‐space of SAO:0.01Mn^4+^ (red) and SAO:0.01Mn^4+^,0.1Ga^3+^ (blue).

We measured Mn K‐edge XAFS (X‐ray absorption fine structure) for obtaining information of local structure and property around Mn^4+^. **Figure** **6**a shows EXAFS (extended X‐ray absorption fine structure) oscillation and XANES (X‐ray absorption near edge structure).^[^ ^19^ ^]^ Figure 6a exhibits the difference in EXAFS oscillation between SAO:Mn^4+^ and SAO:Mn^4+^,Ga^3+^. In Figure 6b, Fourier transform analyzed from Mn K‐edge EXAFS oscillation exhibits the 1st nearest peak (Mn–O) and the 2nd nearest peak (Mn–(O)–Al or Mn–(O)–Ga). The average distance in Mn–O peak is quite similar in between SAO:Mn^4+^ and SAO:Mn^4+^,Ga^3+^, which is consistent with the result of Rietveld analysis, while the average distance in Mn–(O)–Al/Ga peak of SAO:Mn^4+^,Ga^3+^ is significantly larger than that in Mn–(O)–Al average distance of SAO:Mn^4+^, which is an evidence that Ga makes bond with [MnO_6_] octahedron. Figure S4 in the Supporting Information shows tall and sharp edge of Mn K‐edge XANES, where the edge of SAO:Mn^4+^,Ga^3+^ a little shifts towards higher energy level compared with that of SAO:Mn^4+^, which is also an evidence of Mn–(O)–Ga because Ga^3+^ having relatively higher electronegativity *χ* (Pauling's *χ*(Ga): 1.81) reasonably lowers electron density of Mn^4+^ compared with Al^3+^ (*χ*(Al): 1.61) through Mn–(O)–Ga bond.

Figure 6a shows small pre‐edge peak in XANES region, which corresponds to 1s→3d parity forbidden transition of Mn. From the same discussion as that of excitation spectra concerning 3d→3d parity forbidden transition, Mn^4+^ on Al_4_ site having no inversion symmetry (site symmetry in O_h_: C_2_) mainly contributes to both ^2^E→^4^A_2_ transition (red emission) and 1s→3d transition (pre‐edge X‐ray absorption peak) rather than Mn^4+^ on Al_5_ or Al_6_ site having inversion symmetry (site symmetry in O_h_: C_2h_).^[^ ^20^ ^]^

As shown in Figure 6a, we observed that pre‐edge peak of SAO:Mn^4+^,Ga^3+^ is significantly larger than that of SAO:Mn^4+^ (about 80% increase). The degree of increase in the intensity under the substitution of Ga^3+^ coincides in between the pre‐edge peak (1s→3d) and the emission peak of deep red (^4^A_2_→^4^T_2_ and ^2^E→^4^A_2_ in 3d). The XAFS local information of SAO:0.01Mn^4+^ and SAO:0.01Mn^4+^,0.1Ga^3+^ is shown in Figure S4 in the Supporting Information.

**Figure 7:**
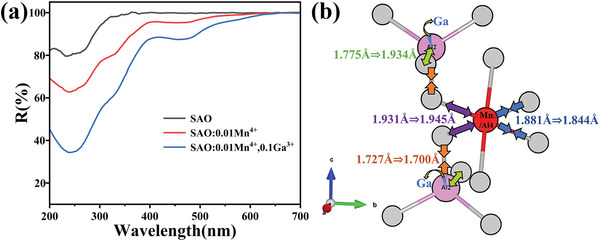
a) SAO Series Reflectance Spectrogram, and b) local structure including Mn^4+^ site and Ga^3+^ site in SAO:Mn^4+^,Ga^3+^.

Parity forbidden transition is analyzed in the next. Electric dipole transition probability *P* is proportional to |∫*Φ*_f_ *HΦ*_i_d*τ*|^2^, where *H* is electric dipole moment *H* = *e* ***ｒ***, *Φ*_i_ and *Φ*_f_ are wave functions of initial and final states, respectively, and d*τ* is d*x*d*y*d*z*.^[^ ^21^ ^]^ For example, ∫*Φ*_f_ *HΦ*_i_d*τ* can be divided into two regions, +*x* region and −*x* region. In case of inversion‐symmetric [Mn^4+^O^2−^_6_] octahedron, an example is shown that *φ*_i_ is d*_x_*_2−_ *_y_*_2_ and *φ*_f_ is d*_z_*_2_, where the sum of the value ∫_−∞_^0^ *Φ*_f_ *e* ***r*** *φ*_i_ dx in −*x* region and ∫_0_^∞^ *Φ*_f_ *e* ***r*** *φ*_i_ d*x* in *x* region is zero due to that parities of *φ*_i_ and *φ*_f_ are even functions (*φ*_i_(*x*) = *φ*_i_(−*x*) and *φ*_f_(*x*) = *φ*_f_(−*x*)). The other combination of two orbitals within (d*_xy_*, d*_yz_*, d*_zx_*, d*_x_*_2−_ *_y_*_2_, and d*_z_*_2_) as *φ*_i_ and *φ*_f_ also provides zero value of transition probability after the equation of transition probability is divided into +*x* region, −*x* region, +*y* region, −*y* region, +*z* region, and −*z* region, and the summation is done in the same way. However, when the bond length is different in between +*x* region and −*x* region, the value ∫_−∞_^0^ *Φ*_f_ *e* ***r*** *φ*_i_ d*x* in −*x* region is not equal to − ∫_0_^∞^ *Φ*_f_ *e* ***r*** *φ*_i_ dx in x region, resulting in that the transition probability is not zero but positive. We found that our Sr_4_Al_14_O_25_: Mn^4+^,Ga^3+^ is in this case, which is shown in **Figure** **7**b. In Sr_4_Al_14_O_25_:Mn^4+^,^[^ ^20^ ^]^ the site symmetry of Al_4_ site occupied by Mn^4+^is C_2_, namely, noninversion symmetry, while the site symmetry of Al_5_ and Al_6_ sites is C_2h_, namely, inversion symmetry (Figure 7b). According to our Rietveld refinement of our Sr_4_Al_14_O_25_:Mn^4+^,Ga^3+^ of which structural parameters of unit cell is shown in Table S1 in the Supporting Information, in −*x* region of MnO_6_ octahedron, the two bonds are elongated (1.931 Å→1.945 Å) due to the shrink (1.727 Å→1.700 Å) of nearby bond by substituting Ga^3+^ for [AlO_4_] (Al: Al_2_ site) tetrahedron site. Then, the two bonds in opposite side (+*x* region) in MnO_6_ is reversely reduced (1.881 Å→1.844 Å). Then, the symmetry of d‐orbitals of Mn^4+^ hybridized by p‐orbitals of O^2−^ in between +*x* region and −*x* region is broken, changing from almost parity forbidden transition into parity allowed transition,^[^ ^22^ ^]^ causing much increase of excitation and emission. This breakthrough was fortunately found because Ga^3+^ is sit in Al_2_ site nearby MnO_6_ at Al_4_ site, which was able to be observed by using AC‐STEM (Figure 5), where stronger white circle Ga compared with darker white Al can be seen (Cf. electron number of Ga^3+^ is much larger, 28, than that of Al^3+^, 10, leading to much larger electron scattering cross section.) Breaking almost parity forbidden transition was verified by measuring pre‐edge peak in Mn K‐edge XANES (Figure 6a). Pre‐edge peak (non‐single peak corresponding to almost symmetric *O*_h_ (almost parity forbidden transition of 1s→3d)^[^ ^23^ ^]^ of Mn^4+^ in Sr_4_Al_14_O_25_ was observed to be much increased by substituting Ga^3+^ (about 40% increase). Figure 7a shows the diffused reflection spectra of SAO:Mn^4+^ and SAO:Mn^4+^,Ga^3+^. It is clear that the substitution of tiny amount of Ga^3+^ much increased absorption (^4^A_2_→^4^T_1_ (NUV) and ^4^A_2_→^4^T_2_ (blue)) in the region of UV and blue. It strongly suggests that Ga^3+^ much increases the excitation transition of Mn^4+^, which is consistent with a break of parity forbidden transition under an existence of Ga^3+^.

For a purpose of evaluating the effect of Ga^3+^, we tried to introduce cation different from Ga^3+^ to SAO:Mn^4+^,Mg^2+^. Single phase of SAO:0.014Mn^4+^,0.007Mg^2+^,0.1Z^3+^ (Z: Sc^3+^ or Lu^3+^) was successfully synthesized, whose relative emission intensity is shown in **Table** **1**. The emission increase or decrease in case of Sc^3+^ and Lu^3+^ was only +4% and −8%, respectively, much different from the case of Ga^3+^. Table 1 shows the negligible effect of Mg^2+^ in 1s→3d transition in pre‐edge peak of XANES and the electronegativity difference between M and O for M–O bond (M: Mg, Lu, Sc, and Ga) too. As understood from Table 1, only Ga^3+^ yields large effect of breaking parity forbidden transition, while other cations such as Mg^2+^, Lu^3+^, and Sc^3+^ have negligible effect. When electronegativity difference between M and O is small, M–O bond has a covalent character where d orbital of M cation is significantly hybridized by p orbital of O anion. It was reported^[^ ^24^ ^]^ that in perovskite cuprates La_2_CuMO_6−_ *_x_* (M′: Mn, Ru), Cu–O bond in CuO_6_ octahedron is much distorted by nearby Ru–O bond having much large electronegativity difference between Ru and O, while CuO_6_ octahedron is not distorted by nearby Mn–O bond having less electronegativity difference between Mn and O, which indicates 3d(Cu)‐2p(O)‐4d(Ru) orbital hybridization is promoted, leading to the distortion of CuO_6_ octahedron by the orbital‐overlapping power of strong covalency of Ru–O bond. The existence or non‐existence of the break of parity forbidden transition in MnO_6_‐M (M: Mg, Sc, Lu, or Ga) in our case is consistent with that of distortion of CuO_6_ octahedron in CuO_6_‐M′ (M: Mn or Ru). It is strongly estimated that only Ga–O bond having significant covalency in a group of Mg, Lu, Sc, and Ga can strongly change the overlapping of 2p orbital in M–O–Mn bonds, distorting MnO_6_ octahedron and breaking parity forbidden d–d transition in Mn^4+^.

**Table 1:** Intensities of deep red emission and pre‐edge peak in Mn‐K XANES, and electronegativity difference of M–O bond (M: Mg, Sc, Lu, and Ga) in Sr_4_Al_14_O_25_:0.014Mn^4+^,0.007X^2+^,0.1Z^3+^

Sr_4_Al_14_O_25_:0.014Mn^4+^,0.007X^2+^,0.1Z^3+^		Relative intensity of deep red emission (3d→3d transition)	Relative intensity of pre‐edge peak in Mn‐K XANES (1s→3d transition)	Electronegativity difference Δ*χ* (*χ*(O)−*χ*(M)) in M–O bond
X^2+^	Z^3+^			
none	none	–	100	–
Mg^2+^	none	100	100	2.13(Mn–O)
Mg^2+^	Sc^3+^	104	–	2.08(Sc–O)
Mg^2+^	Lu^3+^	92	–	2.17(Lu–O)
Mg^2+^	Ga^3+^	160	180	1.63(Ga–O)

Much increase in emission intensity by the substitution of ion breaking parity forbidden transition has never been reported in traditional phosphors having d–d transition or f–f transition such as many Mn^4+^, Mn^2+^, Cr^3+^, or Eu^3+^‐phosphors important for red‐light plant growth, LED lighting and display, CRT television, fluorescent lamp, plasma display, etc. so far, while this has been reported in self trapped exciton (different emission mechanism) of perovskite.^[^ ^21^, ^25^ ^]^ Comparison between pre‐edge peak in XANES and clarified local structure of activator having parity forbidden transition in phosphors are suggested to be effective in our work.

### 4.4 Further Modification of New SAO:Mn^4+^,Ga^3+^ for Charge Compensation

**Figure 8:**
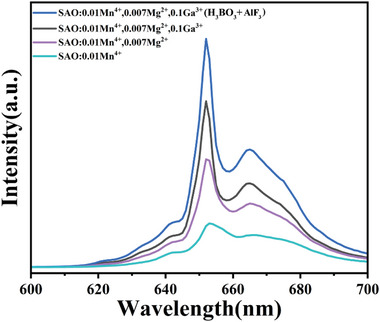
Emission spectra of SAO: Mn^4+^ series after modification.

Possibly, Mn^4+^ activated aluminate has some defects due to a mismatch of electric charge of Mn^4+^ and Al^3+^. Further, we introduced Mg^2+^ because it is strongly expected that a charge balance between Δ+1 (Mn^4+^ on Al^3+^ site) and Δ−1 (Mg^2+^ on Al^3+^ site) can be achieved in the crystal. Figure S8 (Supporting Information) shows the photoluminescence (PL) spectra of the SAO:0.01Mn^4+^,0.1Ga^3+^,*x*Mg^2+^ (0.003 ≤ *x* ≤ 0.009) sample. The emission peak much increased at *x* (Mg molar ratio) = 0.007 without any change of the peak position and ratio of zero phonon line and side bands. Moreover, we found much efficient flux, H_3_BO_3_/AlF_3_ for SAO:Mn^4+^,Mg^2+^,Ga^3+^. **Figure** **8** shows the tremendous feature concerning the emission intensity. Emission intensity of conventional SAO:Mn^4+ [^ ^26^ ^]^ increased +105% by the substitution of 0.007Mg^2+^ due to charge compensation, further increased +60% by the substitution of 0.1Ga^3+^ due to a break of parity forbidden transition for the first time, and further increased +39% by using the new double flux (total increase +355%). The advanced mechanism induced by Ga^3+^ is never induced by Mg^2+^, as shown in Figure 6a, where Mg^2+^ never increases the pre‐edge peak (1s→3d).

## 5 Plant Cultivation

**Figure 9:**
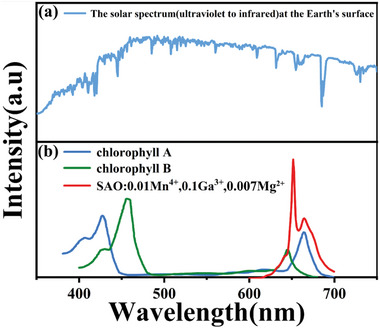
a) Solar spectrum and b) absorption spectra of chlorophyll and emission spectrum of SAO:0.01Mn^4+^,0.1Ga^3+^,0.007Mg^2+^.

Photoconversion films using sunlight for excitation are advanced films that can now be used on a large scale for agricultural growth and have great potential in promoting agricultural development. **Figure** **9**a shows that the blue part of the spectrum (NUV to trans‐infrared) of sunlight irradiated to the earth's surface is the strongest, and the phosphor in this paper has a wide absorption range of NUV–blue light, which makes the SAO: 0.01Mn^4+^, 0.1Ga^3+^,0.007Mg^2+^ powders dispersed PDMS‐film be well excited by sunlight and produce emission spectrum matching well with chlorophyll a chlorophyll b absorption band, as shown in Figure 9b. Accordingly, we conducted experiments on the growth of chlorella vulgaris (nutritious algae) and rose (popular flower).

### 5.1 Experiment of Chlorella Growth

**Figure 10:**
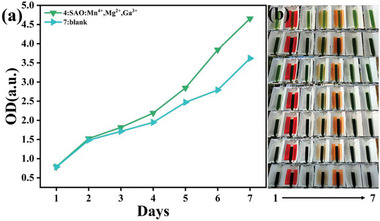
a) Change of optical density of chlorella in tube in a week and b) photograph of chlorella tube and nearby film (SAO:Mn,Mg,Ga film and blank).

Outdoor experiments of chlorella growth were conducted. Sunlight irradiates the light‐conversion film placed at 45°, and the film absorbs blue and NUV light, converting it to red light, which is directed to the nearby glass tube including chlorella, as shown in **Figure** **10**b. Chlorella grows fast and has a short growth cycle of about 7 d. Therefore, chlorella is suitable for verifying the growth promotion effect of the photoconversion film. CO_2_ gas is constantly flown in chlorella tube to meet the photosynthesis of chlorella during growth. Experimental groups of 20 wt% SAO:Mn,Mg,Ga dispersed PDMS‐film (2 mm × 300 mm × 30 mm) having thin white reflector film on its back side were set up as well as a sample having nonphosphor film (simple white reflector) as blank group. The optical density (OD) of chlorella was measured for one week to characterize the growth effect of chlorella. The greater the OD, the higher the concentration of chlorella. Standard deviation of the values was determined by measuring 2 samples kept under the same condition.

After a week‐long experiment, the growth with SAO:Mn,Mg,Ga was 36±14% higher than that with the control group, as shown in Figure 10a. Therefore, our prepared photoconversion film has good practical application prospects for promoting the growth of chlorella vulgaris.

### 5.2 Experiment of Rose Growth

**Figure 11:**
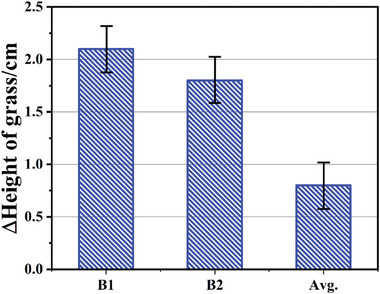
Increase of the height of rose‐grass after 85 d.

In the experiment of rose growth, SAO:Mn,Mg,Ga light conversion films having thin white reflector on its back side were located on both sides of the rose plants and the experiment was conducted for total 85 d during which the variables were strictly checked. Standard deviation of the values was determined by measuring 3 samples kept under the same condition. At the end of the experiment, the growth of height of rose grass was measured concerning 20 wt% SAO:Mn,Mg,Ga/ PDMS film (B1), 10 wt% SAO:Mn,Mg,Ga/ PDMS film (B2), and blank (Avg.). After 85 d‐long experiment, the growth of rose grass was 174±80% (20 wt% SAO) and 125±65% (10 wt% SAO) higher than that of the control group (blank), respectively (**Figure** **11**). Earlier flowering of rose compared with blank was achieved in 20 wt% SAO. A combination of the SAO:Mn,Mg,Ga and the system of reflection‐type film had a significant effect in promoting the growth of rose plants.

## 6 Conclusions

Mn^4+^’s weak excitation and emission due to the property of parity forbidden transition was much increased by an introduction of tiny amount of new cation Ga^3+^ to Mn^4+^‐phosphor. We found 60% increase of red emission of Sr_4_Al_14_O_25_:0.01Mn^4+^ by substituting 0.1Ga^3+^. It was clarified that the increase is originated from a unique mechanism of breaking parity forbidden transition under the substitution of cation in d–d transition by using the tool of AC‐STEM, pre‐edge peak (1s→3d) Mn K‐edge XANES and EXAFS, Rietveld analysis of XRD patterns, and reflection spectra. Substitution of Mg^2+^, Sc^3+^, or Lu^3+^ forming ionic bond with oxygen did not show any tendency of a break of parity forbidden transition. Further, a combination of substituted Ga, Mg, and special double flux H_3_BO_3_/AlF_3_ was found to tremendously increase the emission intensity (355% up). We examined actual growth of chlorella and rose by a combination of the quite cheap Sr_4_Al_14_O_25_:0.01Mn^2+^,0.007Mg^2+^,0.1Ga^3+^ and a unique reflection typed phosphor‐film system as sunlight converting system. Optical density of chlorella and height of rose grass was increased 36±14% and 174±80% compared with nonphosphor‐film, respectively. Earlier flowering of rose was achieved. It is suggested that a combination of advanced cheap phosphor and advanced sunlight converting system (system of well utilizing nature) could solve the subject of highly costing facilities in LED plant factory.

## Conflict of Interest

The authors declare no conflict of interest.

## Supporting information

Supporting Information

## Data Availability

The data that support the findings of this study are available from the corresponding author upon reasonable request.
