# Supplementary material for: Tremendous Acceleration of Plant Growth by Applying a New Sunlight Converter Sr4Al14− x Ga x O25:Mn4+ Breaking Parity Forbidden Transition
Source: Adv Sci (Weinh). 2022 Nov 24;10(2):2204418. doi: 10.1002/advs.202204418 (PMC9839862; doi:10.1002/advs.202204418)
Supplement: Supplementary file 1 — Supporting Information [file ADVS-10-2204418-s001.pdf]

ADVANCED SCIENCE
Open Access

Supporting Information

for *Adv. Sci.*, DOI 10.1002/advs.202204418

Tremendous Acceleration of Plant Growth by Applying a New Sunlight Converter $Sr_4Al_{14-x}Ga_xO_{25}:Mn^{4+}$ Breaking Parity Forbidden Transition

Shichuan Wang, Takatoshi Seto, Bin Liu*, Yuhua Wang*, Cancan Li, Zhengqiang Liu and Haowen Dong*

Supporting Information

Tremendous acceleration of plant growth by applying a new sunlight converter $Sr_4Al_{14-x}Ga_xO_{25}:Mn^{4+}$ breaking parity forbidden transition

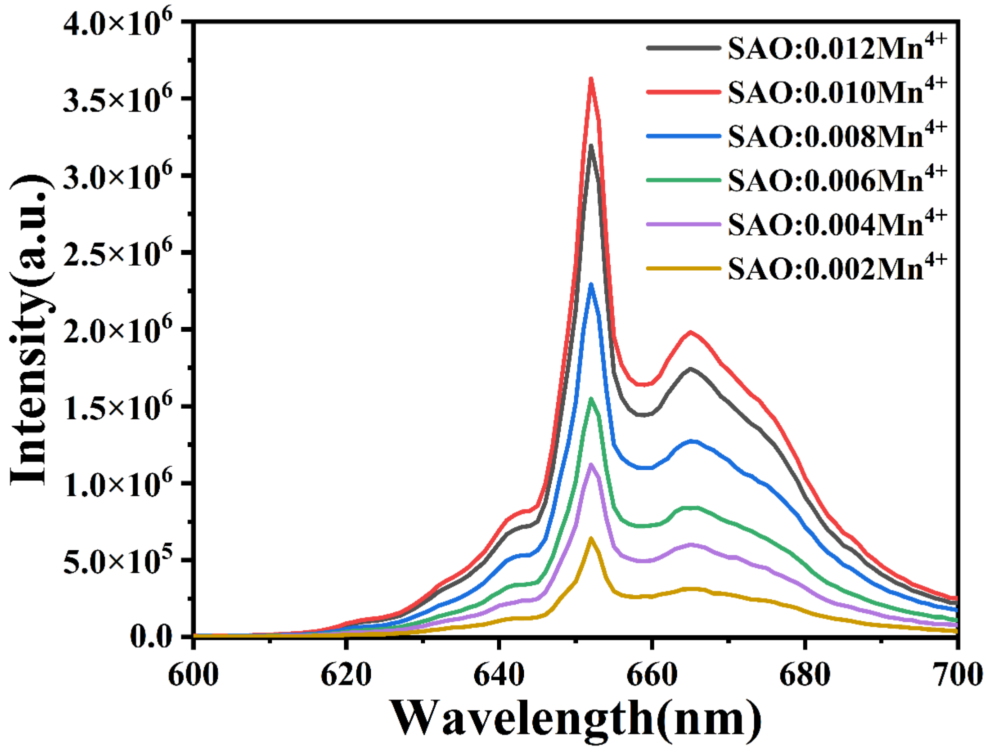

Fig. S1. $Sr_4Al_{14-x}O_{25}:xMn^{4+}$ ($0 \leqslant x \leqslant 0.012$) emission spectrogram.

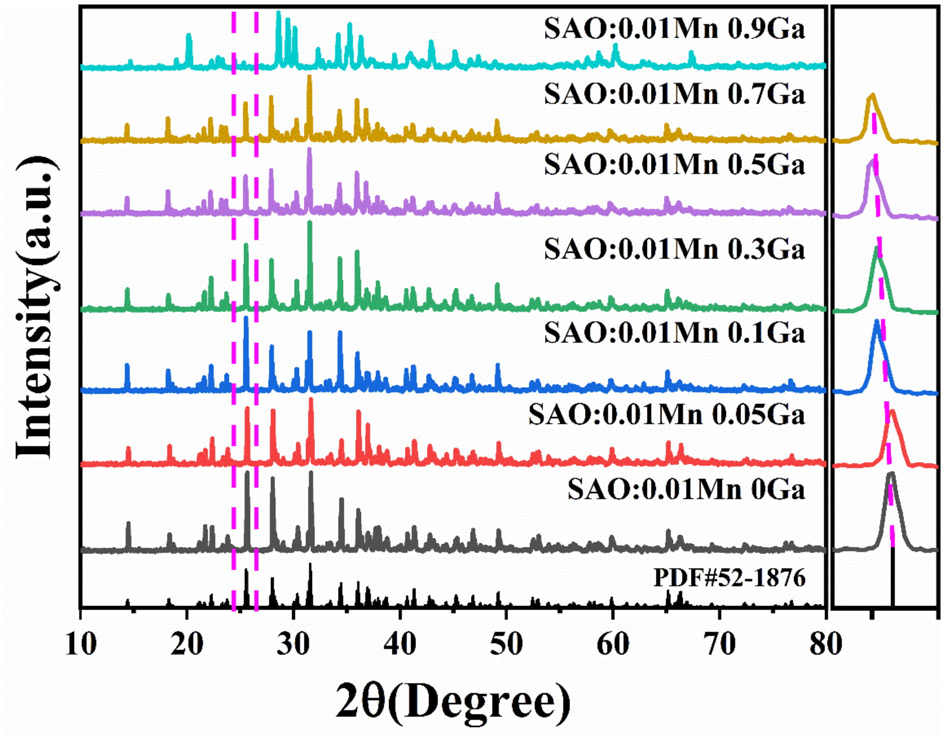

Fig. S2. $Sr_4Al_{14-x}O_{25}:0.01Mn^{4+}$, xGa^{3+} ($0 \leqslant x \leqslant 0.9$) XRD patterns.

Table S1 The atomic coordinate, occupation situation and the average atomic displacement parameter of $Sr_4Al_{14-x}Ga_xO_{25}$ (0≤x≤0.3).

	Atom	Position	Occupancy	x	y	z	Uiso
**$Sr_4Al_{14}O_{25}$ crystal system:orthorhombic Space group: P m m a (51) a = 24.6501(3)Å b = 8.4456(1)Å c = 4.8638(1)Å Rwp =11.82% Rb = 8.90% χ2=1.86 V=1012.57(3) Å^3 Z=2**	Sr1	4j	1.0	0.13720(7)	1/2	0.0416(5)	0.0352(6)
	Sr2	4i	1.0	0.11953(7)	0	0.1115(4)	0.0290(6)
	Al1	8l	1.0	0.14834(22)	0.304(8)	0.6340(12)	0.1195(28)
	Al2	8l	1.0	0.05159(15)	0.3305(5)	0.4711(10)	0.0296(14)
	Al3	4k	1.0	1/4	0.3238(11)	0	0.1445(5)
	Al4	4g	1.0	0	0.1813(6)	0	0.0022(15)
	Al5	2c	1.0	0	0	1/2	0.0249(27)
	Al6	2b	1.0	0	1/2	0	0.085(4)
	O1	8l	1.0	0.04125(20)	0.1617(6)	0.3770(11)	0.0264(18)
	O2	8l	1.0	0.16604(23)	0.2088(7)	0.1048(12)	0.0056(22)
	O3	8l	1.0	0.18159(14)	0.2054(4)	-0.3935(8)	0.0772(12)
	O4	4k	1.0	1/4	0.1605(12)	0.6628(24)	0.096(6)
	O5	4i	1.0	0.04927(29)	0	0.9128(17)	0.0172(26)
	O6	4j	1.0	0.04473(22)	1/2	0.4677(13)	0.0646(19)
	O7	4i	1.0	0.13440(26)	0	0.5772(15)	0.0445(24)
	O8	8l	1.0	0.04090(17)	0.3316(5)	0.8556(10)	0.0496(15)
	O9	2f	1.0	1/4	1/2	0.1454(27)	0.018(6)

	Atom	Position	Occupancy	x	y	z	Uiso
	Sr1	4j	1.0	0.13632(7)	1/2	0.0335(5)	0.0090(6)
	Sr2	4i	1.0	0.12059(8)	0	0.1137(5)	0.0165(6)

**$Sr_4Al_{13.95}Ga_{0.05}O_{25}$ system:orthorhombic Space group: P m m a (51) a = 24.6726(2)Å b = 8.4492(8)Å c = 4.8667(4)Å Rwp =10.78% Rb = 3.69% χ2=2.426 V=1014.549(2) $Å^3$ Z=2**	**Al1**	**8l**	**1.0**	**0.18620(17)**	**0.1947(6)**	**0.6269(11)**	**0.0159(15)**
	**Al2**	**8l**	**0.97**	**0.0648**	**0.33205**	**0.52123**	**0.0176(13)**
	**Al3**	**4k**	**1.0**	**1/4**	**0.2975(7)**	**0.1360(14)**	**-0.0078(20)**
	**Al4**	**4g**	**1.0**	**0**	**0.1762(8)**	**0**	**0.0046(19)**
	**Al5**	**2c**	**1.0**	**0**	**0**	**44563**	**-0.0147(25)**
	**Al6**	**2b**	**1.0**	**0**	**1/2**	**0**	**-0.0092(26)**
	**O1**	**8l**	**1.0**	**0.04418(26)**	**0.1619(10)**	**0.3386(18)**	**0.0487(34)**
	**O2**	**8l**	**1.0**	**0.14372(26)**	**0.2978(11)**	**0.5091(19)**	**0.0458(33)**
	**O3**	**8l**	**1.0**	**0.18855(24)**	**0.2126(10)**	**-0.0271(16)**	**0.0248(28)**
	**O4**	**4k**	**1.0**	**1/4**	**0.2434(19)**	**0.4822(27)**	**0.087(6)**
	**O5**	**4i**	**1.0**	**0.0380(4)**	**0**	**0.8562(25)**	**0.003(4)**
	**O6**	**4j**	**1.0**	**0.04882(33)**	**1/2**	**0.3592(20)**	**-0.0302(32)**
	**O7**	**4i**	**1.0**	**0.1592(4)**	**0**	**0.5566(33)**	**0.083(6)**
	**O8**	**8l**	**1.0**	**0.04080(25)**	**0.3343(10)**	**0.8503(18)**	**0.0379(31)**
	**O9**	**2f**	**1.0**	**1/4**	**1/2**	**0.0559(30)**	**-0.014(5)**
	**Ga**	**8l**	**0.03**	**0.0648**	**0.33205**	**0.52123**	**0.003(15)**

	**Atom**	**Position**	**Occupancy**	**x**	**y**	**z**	**Uiso**
**$Sr_4Al_{13.9}Ga_{0.1}O_{25}$ system:orthorhombic Space group: P m m a (51) a = 24.6879(2)Å b = 8.4555(1)Å c = 4.8714(1)Å Rwp =10.79%**	**Sr1**	**4j**	**1.0**	**0.13730(7)**	**1/2**	**0.0331(5)**	**0.0030(5)**
	**Sr2**	**4i**	**1.0**	**0.12024(7)**	**0**	**0.1163(5)**	**-0.0007(5)**
	**Al1**	**8l**	**1.0**	**0.18637(15)**	**0.1963(5)**	**0.6432(12)**	**0.0030(14)**
	**Al2**	**8l**	**0.94**	**0.06441**	**0.32506**	**0.5057**	**0.0807(24)**
	**Al3**	**4k**	**1.0**	**1/4**	**0.2953(6)**	**0.1251(16)**	**-0.0078(20)**

**Rb = 3.36%**<br>**χ2=2.223**	**Al4**	**4g**	**1.0**	**0**	**0.1646(8)**	**0**	**0.0019(19)**
**V=1016.89(2) Å^3**<br>**Z=2**	**Al5**	**2c**	**1.0**	**0**	**0**	**1/2**	**0.037(4)**
	**Al6**	**2b**	**1.0**	**0**	**1/2**	**0**	**-0.0090(27)**
	**O1**	**8l**	**1.0**	**0.04478(24)**	**0.1610(9)**	**0.3226(20)**	**0.0388(33)**
	**O2**	**8l**	**1.0**	**0.14253(26)**	**0.3090(9)**	**0.5142(19)**	**0.0428(32)**
	**O3**	**8l**	**1.0**	**0.19429(24)**	**0.2254(11)**	**-0.0281(16)**	**0.0417(34)**
	**O4**	**4k**	**1.0**	**1/4**	**0.2315(12)**	**0.4905(19)**	**-0.018(4)**
	**O5**	**4i**	**1.0**	**0.03686(31)**	**0**	**0.8362(24)**	**-0.0187(35)**
	**O6**	**4j**	**1.0**	**0.04997(35)**	**1/2**	**0.3381(26)**	**0.008(4)**
	**O7**	**4i**	**1.0**	**0.16666(34)**	**0**	**0.5884(24)**	**0.003(4)**
	**O8**	**8l**	**1.0**	**0.04401(21)**	**0.3312(8)**	**0.8388(19)**	**0.0012(25)**
	**O9**	**2f**	**1.0**	**1/4**	**1/2**	**0.125(4)**	**0.040(7)**
	**Ga**	**8l**	**0.06**	**0.06441**	**0.32506**	**0.5057**	**-0.0651(29)**

	**Atom**	**Position**	**Occupancy**	**x**	**y**	**z**	**Uiso**
	**Sr1**	**4j**	**1.0**	**0.13810(7)**	**1/2**	**0.0336(5)**	**0.0131(6)**
	**Sr2**	**4i**	**1.0**	**0.11978(7)**	**0**	**0.1140(4)**	**-0.0005(5)**
**$Sr_4Al_{13.9}Ga_{0.3}O_{25}$**<br>**system:orthorhomb**	**Al1**	**8l**	**1.0**	**0.18735(18)**	**0.1954(6)**	**0.6419(12)**	**0.0330(16)**
**ic**<br>**Space group: P m m**	**Al2**	**8l**	**0.82**	**0.06310**	**0.32364**	**0.59617**	**0.170(4)**
**a (51)**<br>**a = 24.6845(3)Å**	**Al3**	**4k**	**1.0**	**1/4**	**0.2840(8)**	**0.1354(16)**	**0.0212(23)**
**b = 8.4544(1)Å**<br>**c = 4.8709(1)Å**	**Al4**	**4g**	**1.0**	**0**	**0.1757(8)**	**0**	**0.0112(20)**
**Rwp =11.07%**<br>**Rb = 4.57%**	**Al5**	**2c**	**1.0**	**0**	**0**	**1/2**	**0.053(4)**
**χ2=2.342**<br>**V=1016.52(2) Å^3**	**Al6**	**2b**	**1.0**	**0**	**1/2**	**0**	**-0.0068(28)**
**Z=2**	**O1**	**8l**	**1.0**	**0.04468(23)**	**0.1595(8)**	**0.3420(17)**	**0.0175(27)**

O2	8l	1.0	0.13761(29)	0.3104(10)	0.4347(16)	0.0551(33)
O3	8l	1.0	0.18874(29)	0.2173(13)	-0.0128(16)	0.103(4)
O4	4k	1.0	1/4	0.253(1)	0.5045(14)	-0.0520(26)
O5	4i	1.0	0.03421(23)	0	0.8271(16)	-0.0755(23)
O6	4j	1.0	0.04549(26)	1/2	0.3842(17)	-0.0619(24)
O7	4i	1.0	0.16646(25)	0	0.5758(16)	-0.0586(23)
O8	8l	1.0	0.04475(23)	0.3229(8)	0.8599(18)	0.0186(27)
O9	2f	1.0	1/4	1/2	-0.132(6)	0.198(13)
Ga	8l	0.18	0.06310	0.32364	0.59617	-0.0900(14)

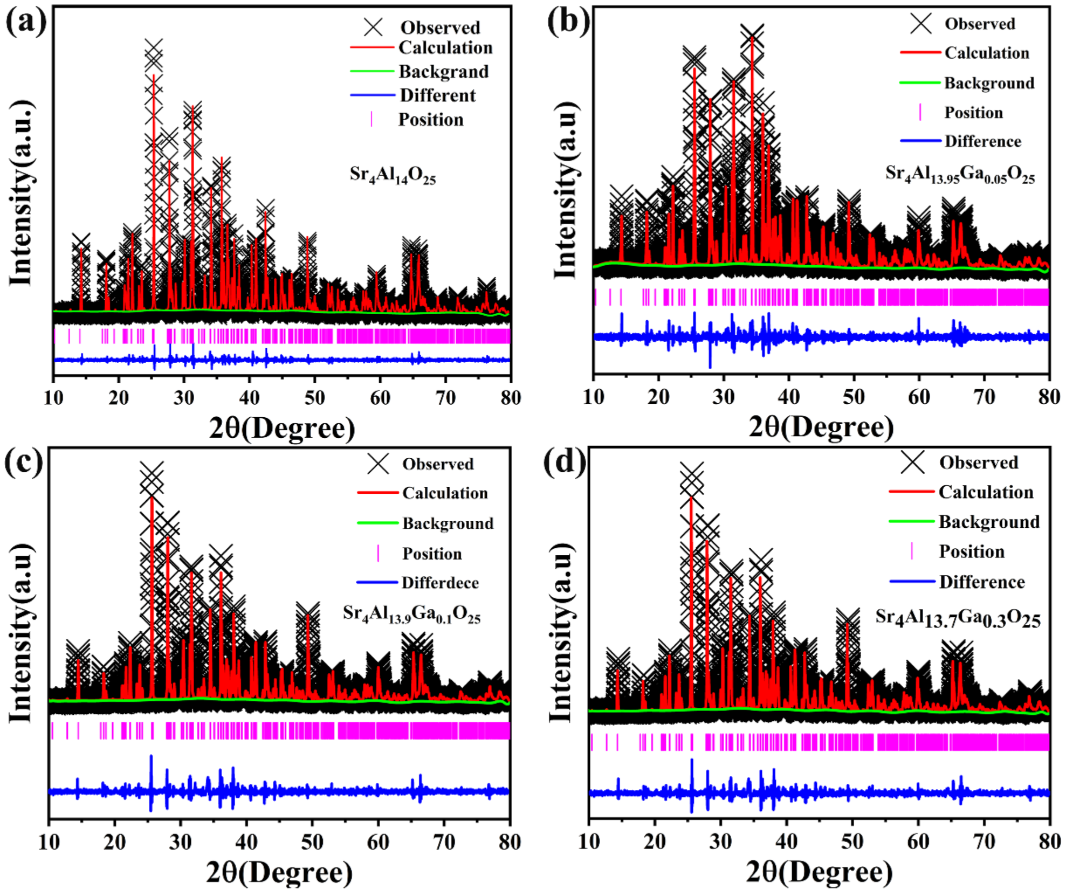

Fig. S3. $Sr_4Al_{14-x}Ga_xO_{25}$ Rietveld diagram.

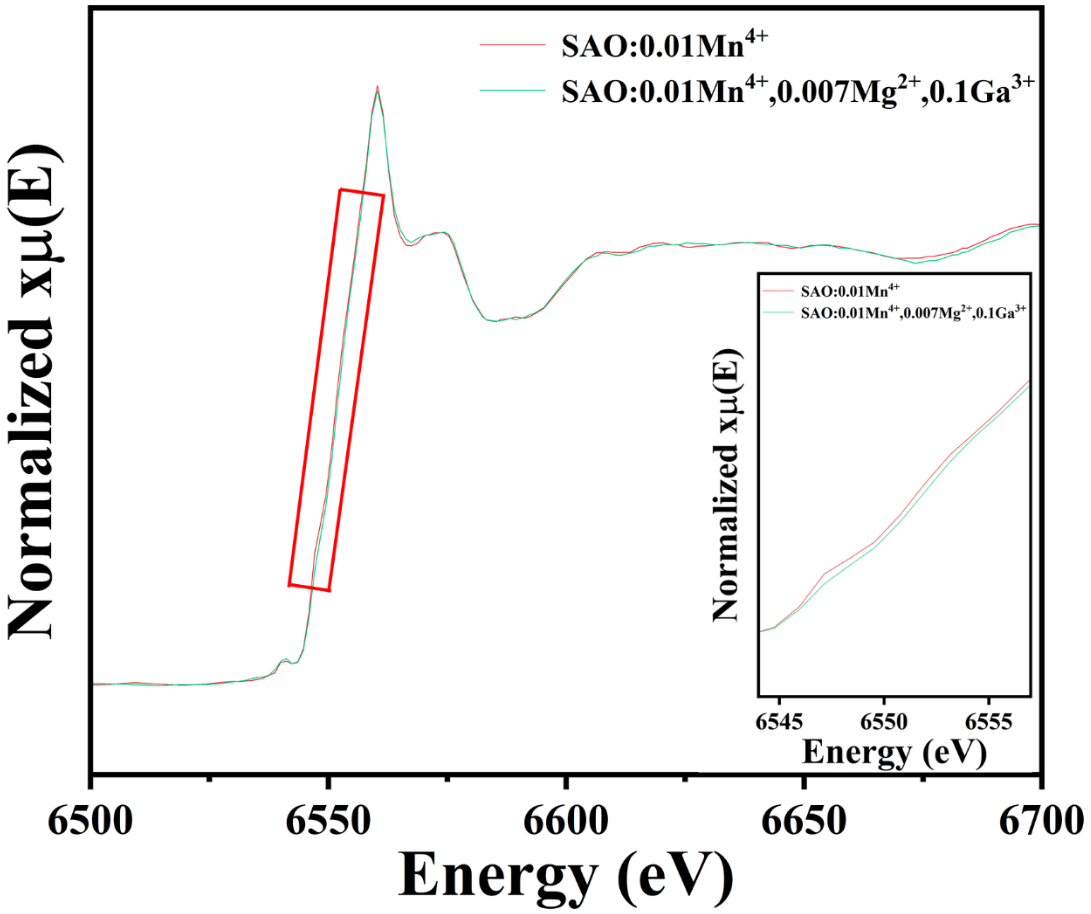

Fig. S4. The XAFS local information of $SAO:0.01Mn^{4+}$ and SAO:0.01Mn4+,$0.1Ga^{3+}$

Table S2. Results of Al-Al distance determined by Rietveld analysis of $Sr_4Al_{14-x}Ga_xO_{25}$.

	Al4-Al4(Å)	Al4-Al5(Å)	Al4-Al6(Å)	Al5-Al6(Å)	Al5-Al5(Å)	Al6-Al6(Å)
$Sr_4Al_{14}O_{25}$	4.86384(8)	2.874(3)	2.692(6)	4.873(6)	8.44556(14)	8.46384(8)
$Sr_4Al_{13.9}Ga_{0.05}O_{25}$	4.88674(8)	2.853(4)	2.736(7)	4.87533(5)	8.44928(10)	4.86674(8)
$Sr_4Al_{13.7}Ga_{0.1}O_{25}$	4.87137(8)	2.805(4)	2.836(7)	4.87919(4)	8.45551(8)	4.87137(8)
$Sr_4Al_{13.5}Ga_{0.3}O_{25}$	4.87091(8)	2.853(4)	2.742(7)	4.8786(5)	8.45441(10)	4.87091(8)

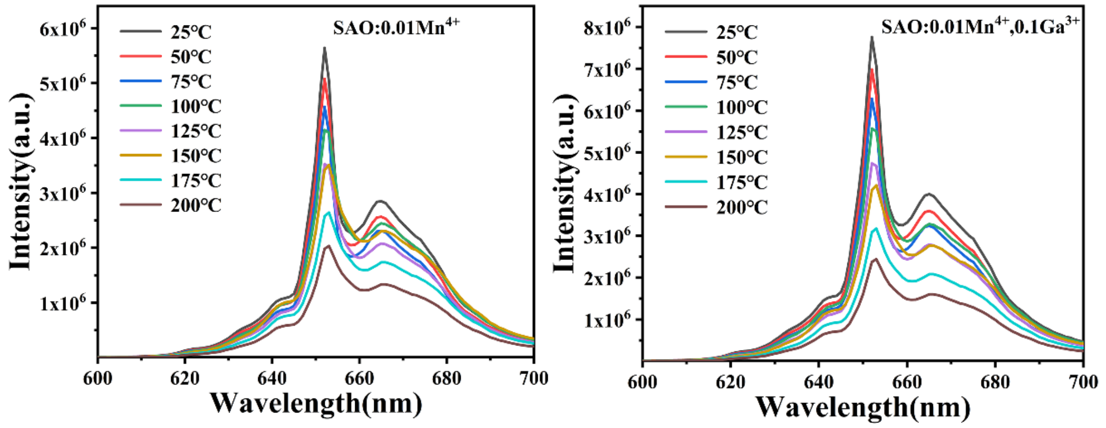

Fig. S5. Thermal burst spectra of $Sr_4Al_{14}O_{25}:Mn^{4+}$， $Sr_4Al_{13.9}O_{25}:0.01Mn^{4+},0.1Ga^{3+}$.

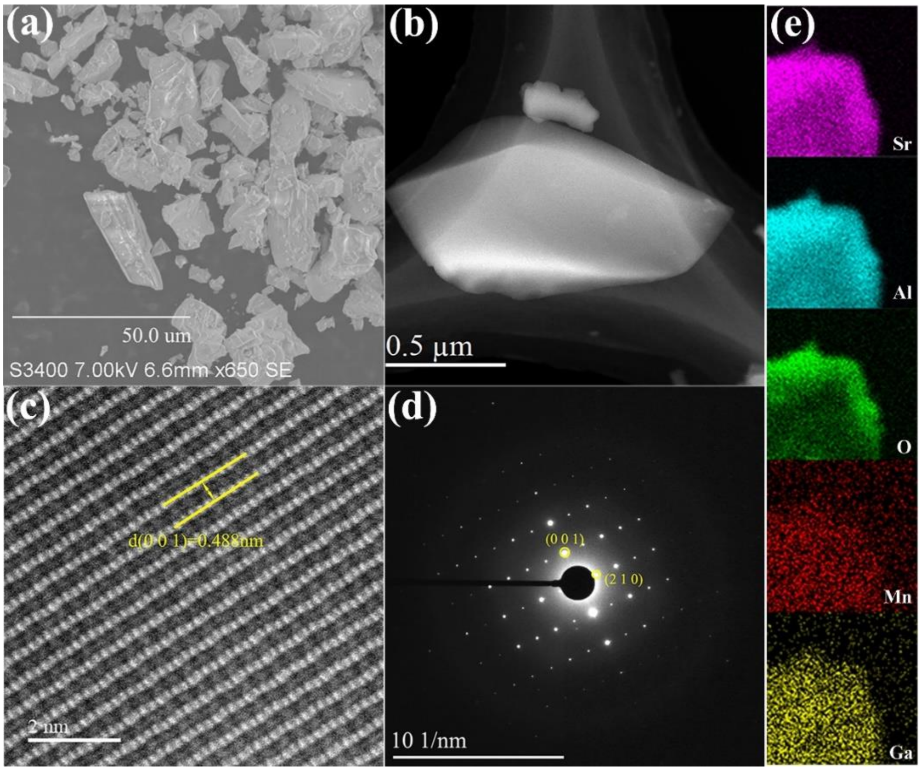

Fig. S6. SEM images of $Sr_4Al_{13.9}O_{25}$:$0.01Mn^{4+}$,$0.1Ga^{3+}$ (a), transmission electron microscopy (b,c,d), EDS (e)

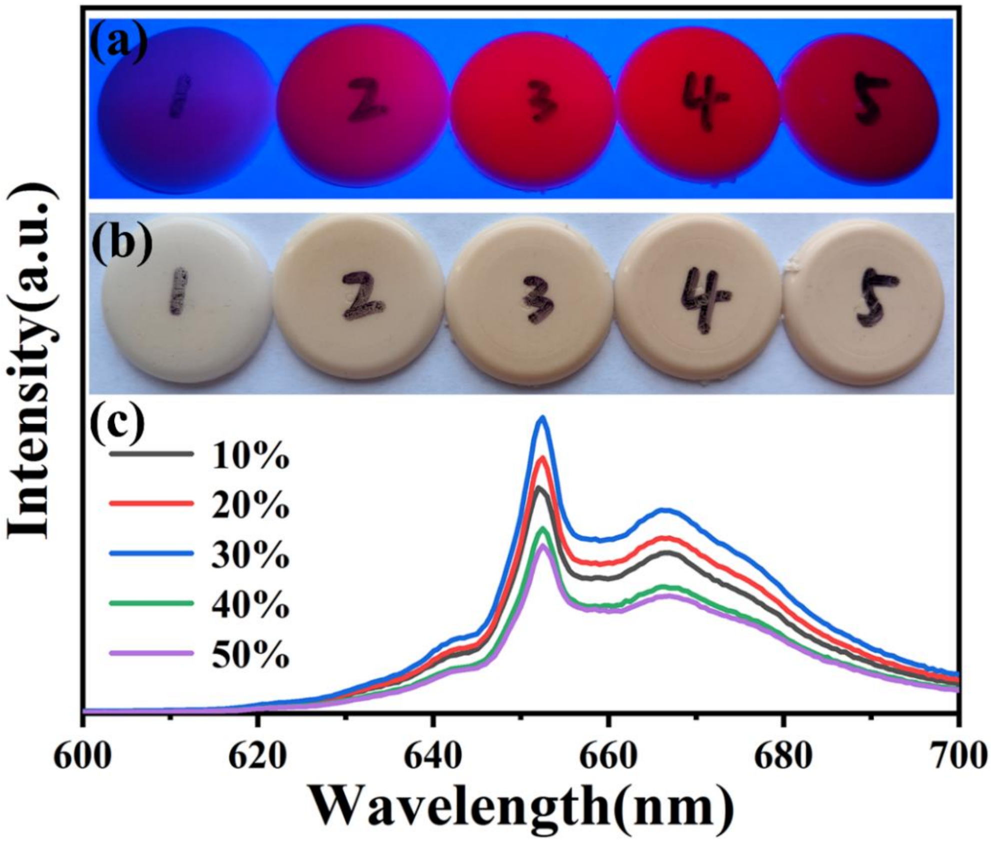

Fig. S7. Phosphor series concentration photoconversion film under UV lamp (a) and under sunlight (b) actual photo and emission spectrum graph (c)

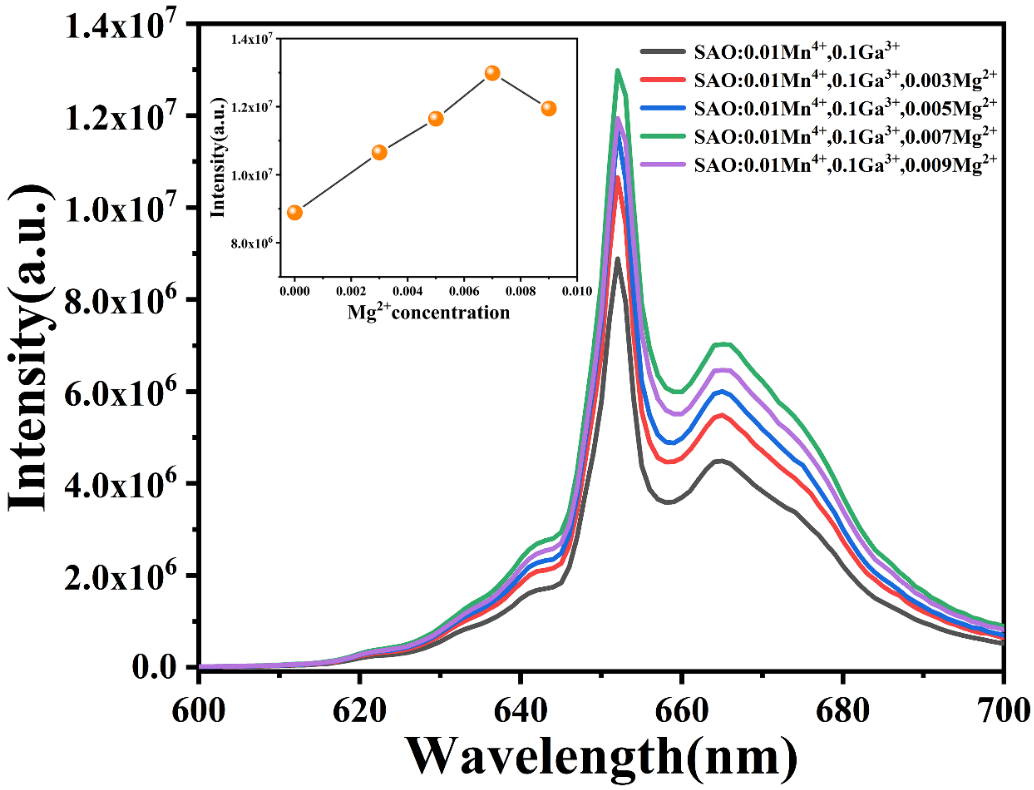

Fig S8. SAO:0.01Mn^{4+},0.1Ga^{3+},xMg^{2+}(0.003≤x≤0.009) emission spectrogram

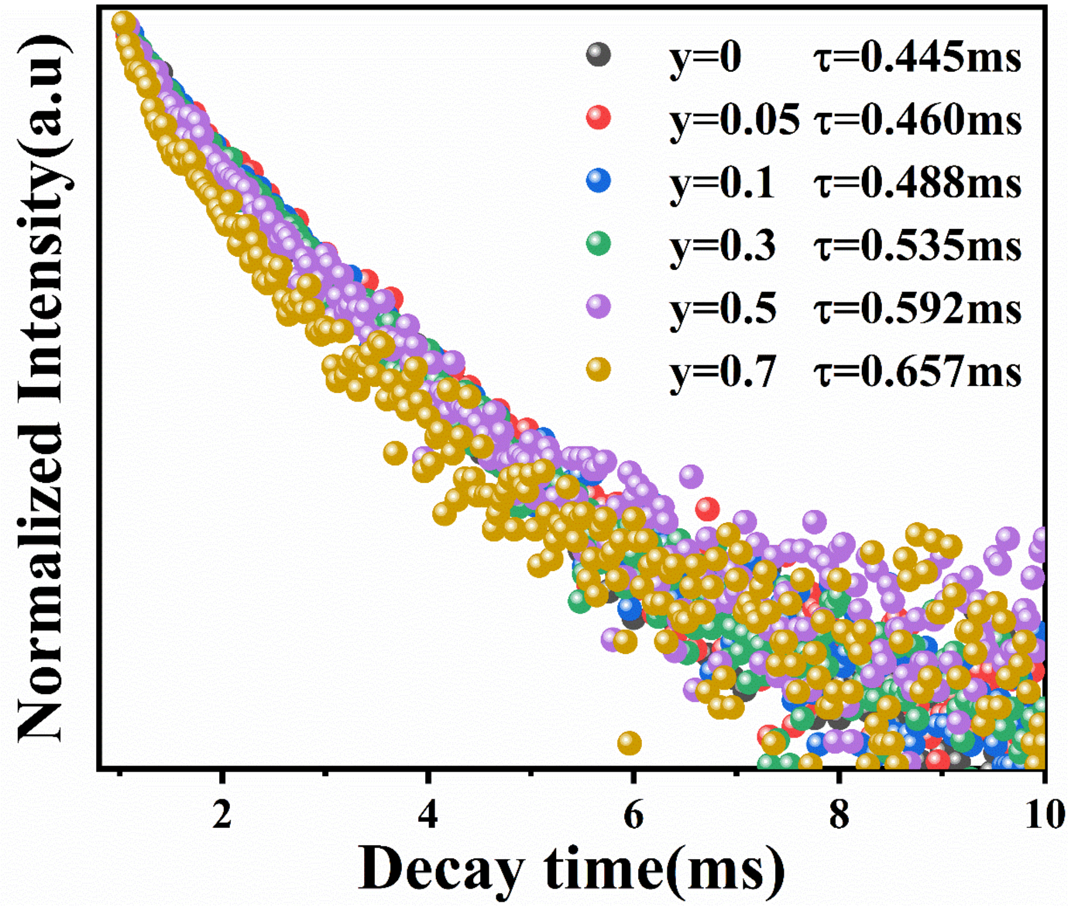

Fig.S9 Series decay of $Sr_4Al_{13.99-y}Ga_yO_{25}$:0.01$Mn^{4+}$

Table S3. The internal quantum efficiencies (IQE) and external quantum efficiencies (EQE) of $Sr_4Al_{13.89}Ga_{0.1}O_{25}$:0.01$Mn^{4+}$

	$\mathbf{Sr_4Al_{13.89}Ga_{0.1}O_{25}:0.01Mn^{4+}}$
**IQE**	49.80%
**EQE**	31.61%
